# Dual Dirac Nodal Line in Nearly Freestanding Electronic Structure of β-Sn Monolayer

**DOI:** 10.1021/acsnano.4c01322

**Published:** 2024-08-01

**Authors:** Ye-Shun Lan, Chia-Ju Chen, Shu-Hua Kuo, Yen-Hui Lin, Angus Huang, Jing-Yue Huang, Pin-Jui Hsu, Cheng-Maw Cheng, Horng-Tay Jeng

**Affiliations:** † Department of Physics, National Tsing Hua University, Hsinchu 30013, Taiwan; ‡ National Synchrotron Radiation Research Center, Hsinchu 30076, Taiwan; § Center for Theory and Computation, National Tsing Hua University, Hsinchu 30013, Taiwan; ∥ Physics Division, National Center for Theoretical Sciences, Taipei 10617, Taiwan; ⊥ Center for Quantum Technology, National Tsing Hua University, Hsinchu 30013, Taiwan; # Department of Electrophysics, National Yang Ming Chiao Tung University, Hsinchu 30010, Taiwan; ¶ Department of Physics, National Sun Yat-sen University, Kaohsiung 80424, Taiwan; ∇ Institute of Physics, Academia Sinica, Taipei 11529, Taiwan

**Keywords:** STM, ARPES, DFT, 2D Dirac nodal line, β-Sn

## Abstract

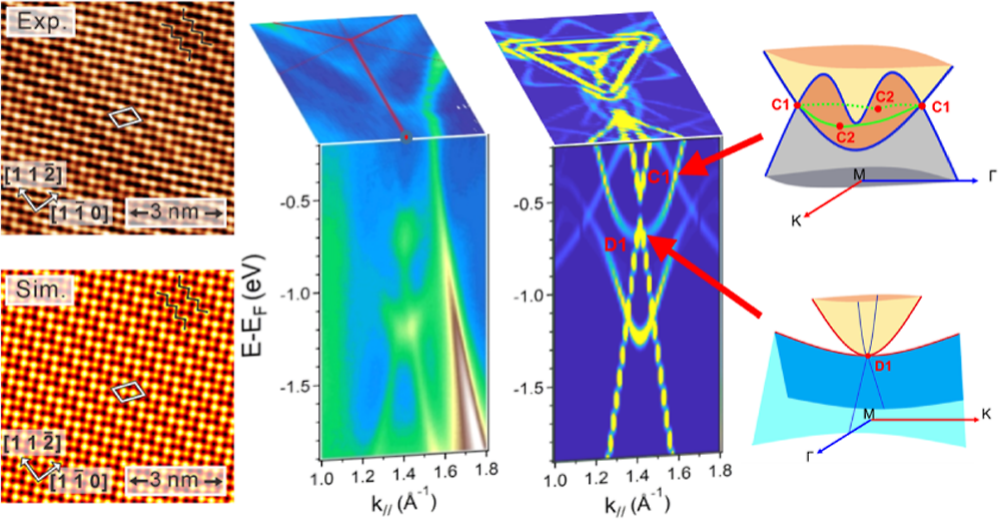

Two-dimensional topological insulators (2D TIs) have distinct electronic properties that make them attractive for various applications, especially in spintronics. The conductive edge states in 2D TIs are protected from disorder and perturbations and are spin-polarized, which restrict current flow to a single spin orientation. In contrast, topological nodal line semimetals (TNLSM) are distinct from TIs because of the presence of a 1D ring of degeneracy formed from two bands that cross each other along a line in the Brillouin zone. These nodal lines are protected by topology and can be destroyed only by breaking certain symmetry conditions, making them highly resilient to disorder and defects. However, 2D TNLSMs do not possess protected boundary modes, which makes their investigation challenging. There have been several theoretical predictions of 2D TNLSMs, however, experimental realizations are rare. β-Sn, a metallic allotrope of tin with a superconducting temperature of 3.72 K, may be a candidate for a topological superconductor that can host Majorana Fermions for quantum computing. In this work, single layers of α-Sn and β-Sn on a Cu(111) substrate are successfully prepared and studied using scanning tunneling microscopy, angle-resolved photoemission spectroscopy, and density functional theory calculations. The lattice and electronic structure undergo a topological transition from 2D topological insulator α-Sn to 2D TNLSM β-Sn, with two types of nodal lines coexisting in monolayer β-Sn. Such a realization of two types of nodal lines in one 2D material has not been reported to date. Moreover, we also observed an unexpected phenomenon of freestanding-like electronic structures of β-Sn/Cu(111), highlighting the potential of ultrathin β-Sn films as a platform for exploring the electronic properties of 2D TNLSM and topological superconductors, such as few-layer superconducting β-Sn in lateral contact with topological nodal line single-layer β-Sn.

## Introduction

Two-dimensional topological insulators (2D TIs) possess distinct electronic properties that make them attractive for a variety of applications, particularly in the field of spintronics.^1−6^ A key feature of 2D TIs is the presence of protected metallic edge states that are immune to disorder and perturbations. These conductive edge states in 2D TIs are spin-polarized, which means that the current flow is restricted to a single spin orientation. This property makes these materials suitable for applications in quantum computing electronics. In a 2D TI, a topological band gap induced by strong spin–orbit coupling (SOC) provides a measure of the insulating behavior of the bulk material and helps to protect the edge states from backscattering and disorder. The size of the topological band gap is an important factor in determining the magnitude of the quantized Hall conductivity,^7,8^ as well as the robustness of the quantum anomalous Hall effect (QAHE) against disorder and other perturbations. A larger topological band gap indicates a stronger insulating behavior of the system, which in turn leads to a more pronounced QAHE.^9,10^ Additionally, topological nodal line semimetals (TNLSM) are distinct from TIs due to the presence of topological nodal lines—a one-dimensional ring of degeneracy formed from two bands that cross each other along a line in the Brillouin zone (BZ).^11^ These nodal lines are protected by topology and can be destroyed only by breaking certain symmetry conditions, which makes them highly resilient to disorder and defects. Although there are a few 2D materials that have been predicted to display topological nodal line or nodal line semimetal (NLS) behavior,^12−15^ experimental realization of 2D TNLSM materials are very rare. Recent studies on 2D transition metal chalcogenides show nodal line band structures in CuSe^16^ and AgSe^17^ with the same honeycomb lattice structure, while Dirac nodal line (DNL) is reported in monolayer (ML) Cu_2_Si in hexagonal lattice structure.^18^ Owing to the similar structural character, all three materials show the same type-I nodal line behavior with concentric loops centered around the Γ point.

Stanene, also known as single layer α-Sn, consists of Sn atoms and forms a buckled honeycomb crystal structure. It is a 2D topological insulator with an inverted band gap of approximately 0.3 eV due to strong SOC.^19^ This is in contrast to silicene and germanene, which have predicted band gaps of approximately 2 and 30 meV, respectively, owing to the weak SOC.^20^ The 0.3 eV topological band gap of stanene makes it a promising 2D material for exploring the quantum spin Hall effect and QAHE at room temperature.^5^ The buckling structure of stanene allows for overlapping between σ and π orbitals, leading to mixing of the sp^2^ and sp^3^ orbitals. The band structure of α-Sn can be tuned with applied strain. Several studies apply strains by using various substrates to modulate the band structure of α-Sn. For example, a few-layer α-Sn grown on InSb(001) and InSb(111) shows a topological transition under a slight in-plane compressive strain due to the lattice mismatch with the substrate.^21−25^ Stanene has also been grown on various substrates such as PdTe(111),^26,27^ Bi_2_Te_3_,^28^ Au(111),^29^ Ag(111),^30^ Sb(111)^31^ and Cu(111),^19^ suggesting a capability to tune the electronic structure of stanene with strain engineering. Moreover, epitaxial growth of stanene on Cu(111) has demonstrated an in-plane s–p band inversion with a topological gap of approximately 0.3 eV induced by SOC at the Γ point,^19^ which is an important property for the realization of topological electronic devices.

β-Sn is a metallic allotrope of tin with a body-centered tetragonal crystal structure that is more stable at high temperatures. It is also one of the earliest superconductors, with a superconducting temperature of 3.72 K.^32−34^ Few-layer superconducting Sn^26^ in conjunction with topological 2D materials such as single layer nodal line β-Sn presented in this work can be a good candidate as a 2D topological superconductor to host Majorana Fermions for quantum computing. Unlike single-layer α-Sn, there have been few studies of the electronic structure for β-Sn ultrathin films to date. Previous reports^35−37^ suggested that the cubic Sn films with fourfold symmetric structures correspond to bilayer β-Sn(001).

In this work, we used scanning tunneling microscopy (STM), angle-resolved photoemission spectroscopy (ARPES), and density-functional theory (DFT) calculations to explore the electronic structure of single layer β-Sn. We first prepared a single layer of α-Sn on a Cu(111) substrate at a low temperature. STM and low energy electron diffraction (LEED) patterns revealed a honeycomb lattice of stanene grown on Cu(111) with the *p*(2 × 2) supercell, and the measured band structure by ARPES showed consistency with reported results.^19,38,39^ We then deposited the same amount of Sn atoms onto stanene/Cu(111) at low temperature, and the honeycomb lattice of stanene/Cu(111) disappeared. Instead, a single layer high-coverage Sn (HC-Sn), cubic β-Sn, phase was observed in the STM images. The observed structural phase transition from semiconducting honeycomb α-Sn to metallic cubic β-Sn presents an opportunity to investigate the topological transition in the band structure of ultrathin β-Sn(001) thin films. Excellent agreement between ARPES spectra and DFT band structures reveals the nearly free-standing electronic structure as well as the topological transition of ML β-Sn, which displays two types of nodal line coexisting in a 2D topological semimetal not reported to date. This discovery highlights the potential of ultrathin β-Sn films as a platform for exploring 2D TNLSM and 2D topological superconductors.

## Results and Discussion

**Figure 1:**
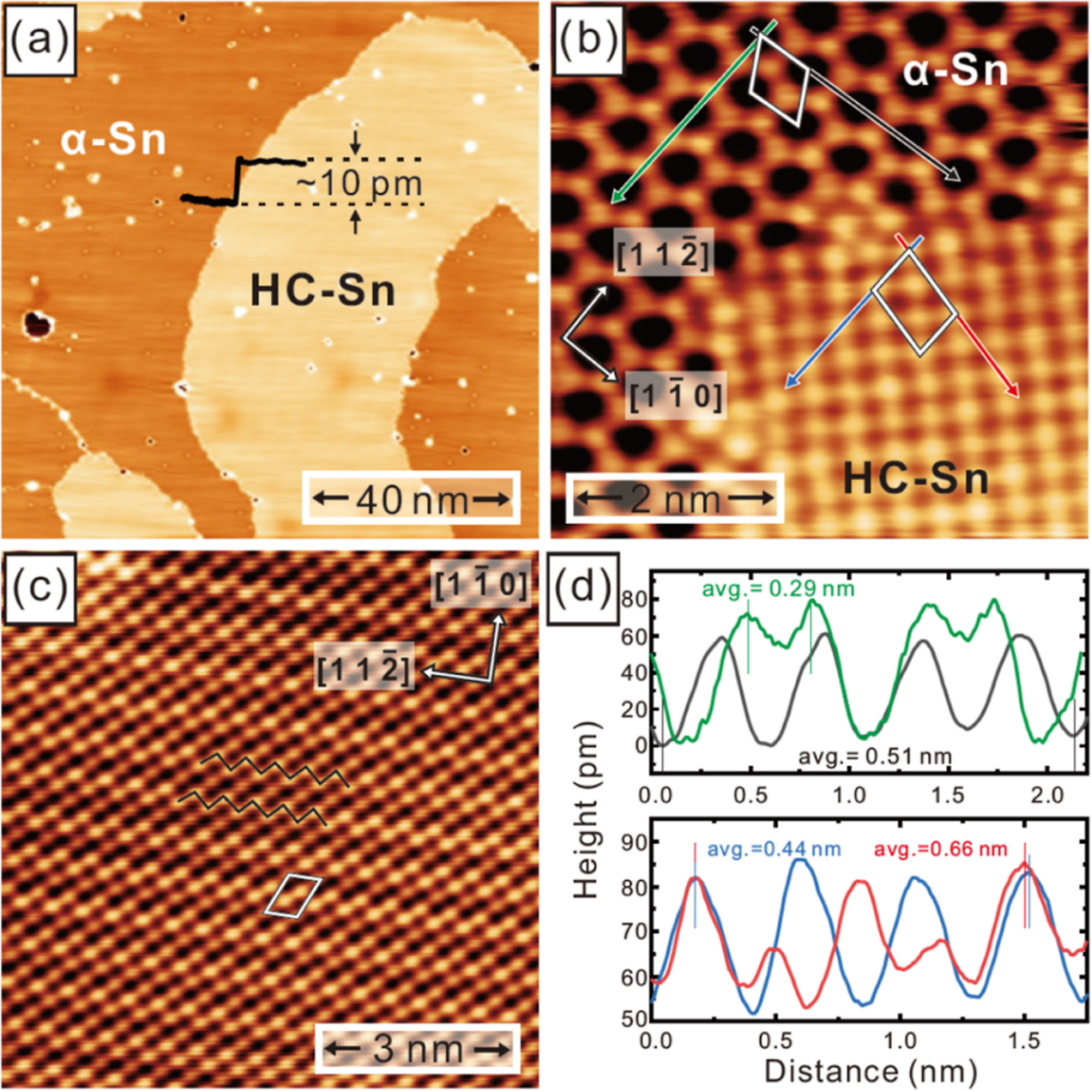
(a) STM topographic overview of about 0.54 ML of Sn grown on Cu(111). The apparent height difference between α-Sn and HC-Sn is about 10 pm (scan parameters: *U* = +1.0 V, *I* = 0.4 nA). (b) Atomically resolved image at the boundary between α-Sn and HC-Sn (scan parameters: *U* = +0.2 V, *I* = 1.0 nA). The white rhombuses indicate the unit cells of α-Sn [*p*(2 × 2)] and HC-Sn. (c) STM image of HC-Sn on a larger scan area with atomic resolution. The periodic zigzag pattern (black zigzag stripes) has been clearly resolved. White rhombus frame denotes the unit cell (scan parameters: *U* = +1.0 V, *I* = 1.0 nA). (d) Top panel: black and green curves are topographic line profiles taken along the arrow lines on the α-Sn phase along [11̅0] and [112̅] directions, respectively, in (b). Bottom panel: topographic line profiles (red and blue curves) taken along the arrow lines on HC-Sn in (b).

Figure 1a shows the STM topographic overview of the Sn thin film grown on the Cu(111) substrate held at 80 K. The bright and dark areas represent regions with high and low apparent heights, respectively, where the bright areas are approximately 10 pm higher than the dark areas. Figure 1b provides the zoomed-in atomically resolved image of the boundary region between the bright and dark areas. An ultraflat honeycomb lattice with a *p*(2 × 2) supercell corresponding to the alpha phase of Sn (α-Sn) has been resolved in the left-upper dark area. In contrast, a higher-coverage Sn phase (HC-Sn) with a more closely packed Sn atom arrangement than the α-Sn is observed in the bottom-right bright area with the white rhombus frame indicating the surface unit cell. It is worth noting that the Cu(111) surface is covered by the α-Sn only, if the amount of Sn deposited is less than or equal to 0.5 ML at 80 K. When the deposition amount of Sn exceeds 0.5 ML, the HC-Sn phase then appears on the Cu(111) surface. Here the amount of ML refers to the pseudomorphic growth of single atomic layer on the Cu(111) surface. Figure 1c shows the wide-ranging atomically resolved image of HC-Sn from the bright area of Figure 1a. The periodic zigzag pattern, as marked by the black zigzag lines resulting from two brighter- and one darker-Sn in the rhombus surface unit cell has been identified.

To determine the lattice constants quantitatively, the topographic line profiles are measured along the high symmetry directions of α-Sn and HC-Sn indicated in Figure 1b. As shown in the top panel of Figure 1d, the average peak-to-peak distances in the α-Sn along [112̅] (green curve) and [11̅0] (black curve) directions are 0.29 and 0.51 nm, respectively, which are in a good agreement with the reported honeycomb lattice of stanene grown on Cu(111) with the *p*(2 × 2) supercell.^19,39^ Based on Figure 1b and the bottom panel of Figure 1d, the extracted lattice constants (white rhombus frame) of the HC-Sn phase are 0.44 and 0.66 nm along [11̅0] (blue line) and [112̅] (red line), respectively, which corresponds to  and  times the lattice constant of Cu(111) substrate, respectively.

**Figure 2:**
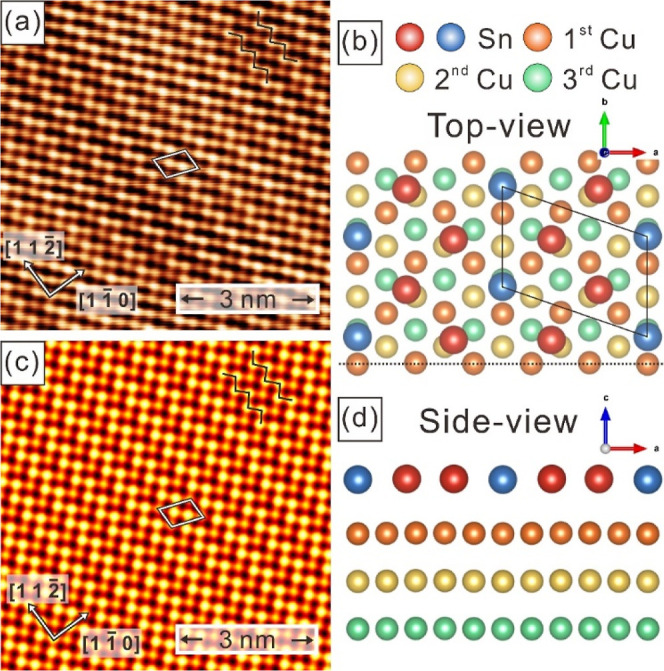
(a) Atomically resolved STM image of (2113) HC-Sn on a larger scan area, where white rhombus frame marks the surface unit cell, and black zigzag stripes indicate the periodic zigzag pattern (scan parameters: *U* = +1.0 V, *I* = 1.0 nA). (b) Top view of the fully relaxed structure model from DFT calculations. The black rhombus indicates the  β-Sn supercell corresponding to the (2113) HC-Sn in (a). The two central Sn (red) sites are located at the hollow-hcp site on top of the 2nd-layer Cu (yellow). The corner Sn (blue) is located at the hollow-fcc site on top of the 3rd-layer Cu (green). (c) Corresponding simulated STM image based on the structure model from (b). (d) Side view of the DFT relaxed structure model. The DFT-optimized interlayer distance between the top β-Sn layer and the 1st Cu layer is 2.40 Å.

Figure 2a represents the atomic resolution image of HC-Sn/Cu(111) with a different scanning angle with respect to Figure 1c to crosscheck the feature of the zigzag pattern and atomic unit cell in order to avoid possible tip-induced artifacts. The corresponding lattice parameters and the orientation of high symmetry crystalline axes can be determined according to the same analyses from Figure 1b–d. Such that we are able to construct the lattice model with a ML  β-Sn supercell on top of a 6-layer Cu(111) substrate for the DFT calculations. The geometrically optimized structure model from fully relaxed DFT calculations is illustrated in Figure 2b,d. The HC-Sn lattice represented by a matrix notation^40,41^ of  contains three Sn atoms per unit cell with a surface coverage of 0.6 ML (Figure 2a). This matrix notation (2113) declares the lattice structure relative to the Cu(111) substrate. The HC-Sn structure resolved by our STM has an additional Sn atom per unit cell as compared to the results deduced from the LEED measurements.^38^ Moreover, the DFT-optimized interlayer distance between the top β-Sn layer and the first Cu layer is 2.40 Å. In comparison with the DFT-optimized interlayer distance between the top α-Sn layer and the first Cu layer of 2.34 Å calculated in our previous work,^39^ the β-Sn layer would be about 0.1 Å higher than the α-Sn layer. This is in good agreement with our experimental finding that the bright areas are approximately 10 pm higher than the dark areas in Figure 1a.

To validate the proposed structure model, we performed STM-image simulations, as presented in Figure 2c. The atomic lattice structure with three Sn atoms per unit cell along with the characteristic periodic zigzag pattern as indicated by black zigzag lines is well consistent with the experimental results in Figure 2a. We also carried out the STM simulations on the (2113) Sn structure with two Sn atoms per unit cell as previously proposed by LEED measurement,^38^ but the results (Supporting Information Figure S1) are not in line with atomically resolved periodic zigzag image in Figure 2a. The zigzag pattern observed in both the experimental and theoretical STM images (Figure 2a,c) originates from the two bright Sn atoms inside the unit cell (rhombus frame) and the dark Sn at the corner of the unit cell. The notable difference in the brightness of Sn atoms can be understood through analyzing the  β-Sn supercell in Figure 2b,d. The two central Sn atoms (red) locate at the hollow-hcp sites on top of the second-layer Cu atoms (yellow), while the corner Sn atom (blue) locates at the hollow-fcc site on top of the third-layer Cu atom (green). Consequently, it is easier for the hcp-site Sn (red) to hybridize with the second layer Cu than for the fcc-site Sn (blue) to hybridize with the third layer Cu. This leads to the higher/lower contribution to the atomic apparent height of central/corner Sn, forming the zigzag pattern in (2113) HC-Sn as shown in Figure 2a,c. Combining all the above consistent experimental and theoretical evidence, we thus confirm the (2113) HC-Sn phase is indeed the single layer β-Sn.

**Figure 3:**
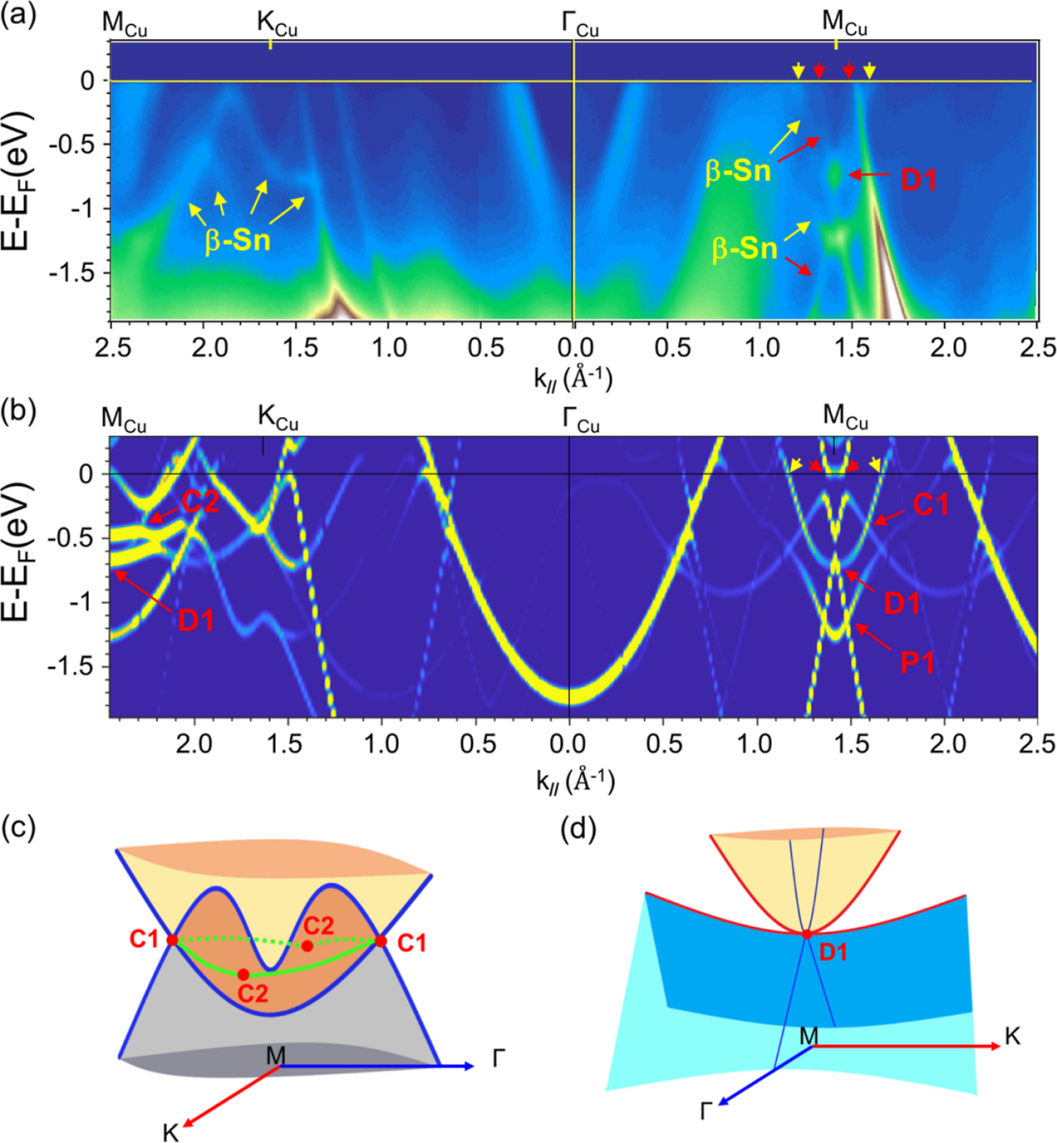
(a) Band dispersions of single layer β-Sn/Cu(111) along M_Cu_–K_Cu_–Γ_Cu_–M_Cu_ relative to the Cu(111) high symmetry point direction. A linear dispersion band with a band crossing D1 located at 0.62 eV appears at the M_Cu_ point. Except the upper and lower Dirac cones, β-Sn derived bands are be labeled with red arrows. (b) Calculated unfolding band structure of freestanding ML β-Sn along the high symmetry lines in the BZ of Cu(111). Schematic diagram of type I (c) and type III (d) nodal line band crossings. C1 and C2 in (c) and D1 in (d) near high symmetry point M_Cu_ are indicated by the red arrows in parts (a) and (b). The dotted and solid green circle in (c) is the nodal ring. In (d), the nodal ring is degraded into one point D1 forming the Dirac-like band crossing. The blue and red parabolas in (c) and (d), respectively, are the energy bands along the M_Cu_–Γ_Cu_ and M_Cu_–K_Cu_ directions.

To deepen the understanding of the electronic structure evolution from low-coverage (LC) to high-coverage (HC) phases of Sn on Cu(111), we performed ARPES measurements to probe the band structures of both phases. The STM studies along with LEED patterns in Figure S2 demonstrate the long-ranged ordered alpha-Sn *p*(2 × 2) supercell relative to Cu(111) substrate below 0.5 ML for the LC phase. The measured band structure of α-Sn/Cu(111) along K′(K_Cu_)–M′–K–Γ–M–Γ′(M_Cu_) direction, with a 0.3 eV topological band gap induced by strong SOC at the Γ′ point (Figure S3), shows the consistency with reported band structure on stanene/Cu(111).^19^Figure S2 also indicates that α-Sn and β-Sn coexist in between 0.5 and 0.6 ML. The sharp LEED patterns above 0.5 ML reveal a phase transition from α-Sn(111) to β-Sn(001) for the HC phase, which is also supported by STM studies in Figure S2 and ARPES results in Figure S3. Figure 3a displays the ARPES band structure of β-Sn/Cu(111), measured along M_Cu_–K_Cu_–Γ_Cu_–M_Cu_ relative to the Cu(111) high symmetry points. In comparison with the band structure of pristine Cu(111) and α-Sn/Cu(111) (Figure S3), the topological band gap at the Γ′ point of α-Sn/Cu(111) disappeared. Instead, additional band structures of β-Sn emerge around the M_Cu_ and K_Cu_ points relative to Cu(111) as discussed below.

In comparison with the band structures of pristine Cu(111) (Figure S3a), a linear band dispersion with a significant Dirac point located at 0.62 eV of binding energy is observed around the M_Cu_ point for β-Sn/Cu(111) (Figure 3a), the band structure suggests an electronic topological transition. For upper Dirac cone, two wave vectors crossing the Fermi level at the red arrows could be clearly extracted from the band mapping result. In addition, two electron pockets around the M_Cu_ point are also observed in Figure 3a. One electron pocket has significant Fermi level crossings labeled by yellow arrows, and the other electron pocket appears in the high binding energy region with the band minimum located at 1.3 eV binding energy. Both electron pockets have band crossings with the lower Dirac cone. Moreover, additional band dispersions originated from β-Sn can be observed at the K_Cu_-point. With a photon energy dependent ARPES experiment, 2D behavior for these band dispersions can be confirmed and attributed to the contribution of β-Sn single layer (Figure S4).

To investigate the nature of topological band dispersion in β-Sn, we performed DFT band structure calculations for both freestanding ML β-Sn and ML β-Sn/6 ML Cu(111) superstructure as described in the Methods section and depicted in Figure S5a. The calculated band structures of β-Sn with/without Cu substrate are presented in Figure S5b–i, which shows highly dispersive metallic bands crossing the Fermi level. To compare with ARPES results, we unfold the band structure in Figure S5 onto the BZ of the Cu(111) 1 × 1 unit cell. The unfolded bands of freestanding ML β-Sn are shown in Figure 3b with the spectral weight indicated by the brightness of the yellow color. The unfolded band structure of the β-Sn/Cu(111) superstructure (Figure S7) is basically the same as the freestanding one (Figure 3b) with minor differences only. Detailed comparison between unfolded band structures of β-Sn with/without Cu substrate are shown in Figures S5–S7.

In comparison with the ARPES in Figure 3a, the unfolded band structure of freestanding β-Sn ML in Figure 3b shows excellent agreement with the ARPES result, especially for the band dispersion around the M_Cu_ and K_Cu_ points. The C1 and D1 band-crossing points around the M_Cu_ point in Figure 3a,b indicated by red arrows can be identified as type I and III NLS behavior^42^ as illustrated in Figure 3c,d, respectively. The C1 crossing point goes through the contour c1–c2–c1–c2–c1 forming a closed-ring-shaped type I nodal line. The band crossing points at C1 and C2 (Figure 3b) show that the two energy bands have opposite slopes in the M_Cu_–K_Cu_ and M_Cu_–Γ_Cu_ directions, and that the two oppositely oriented bowls (concave up and down parabola-shaped bands) in Figure 3c form a type-I Dirac nodal ring. Supporting Information Figure S11 shows detailed energy dispersions and precise locations of the DNL in the 2D BZ.

**Figure 4:**
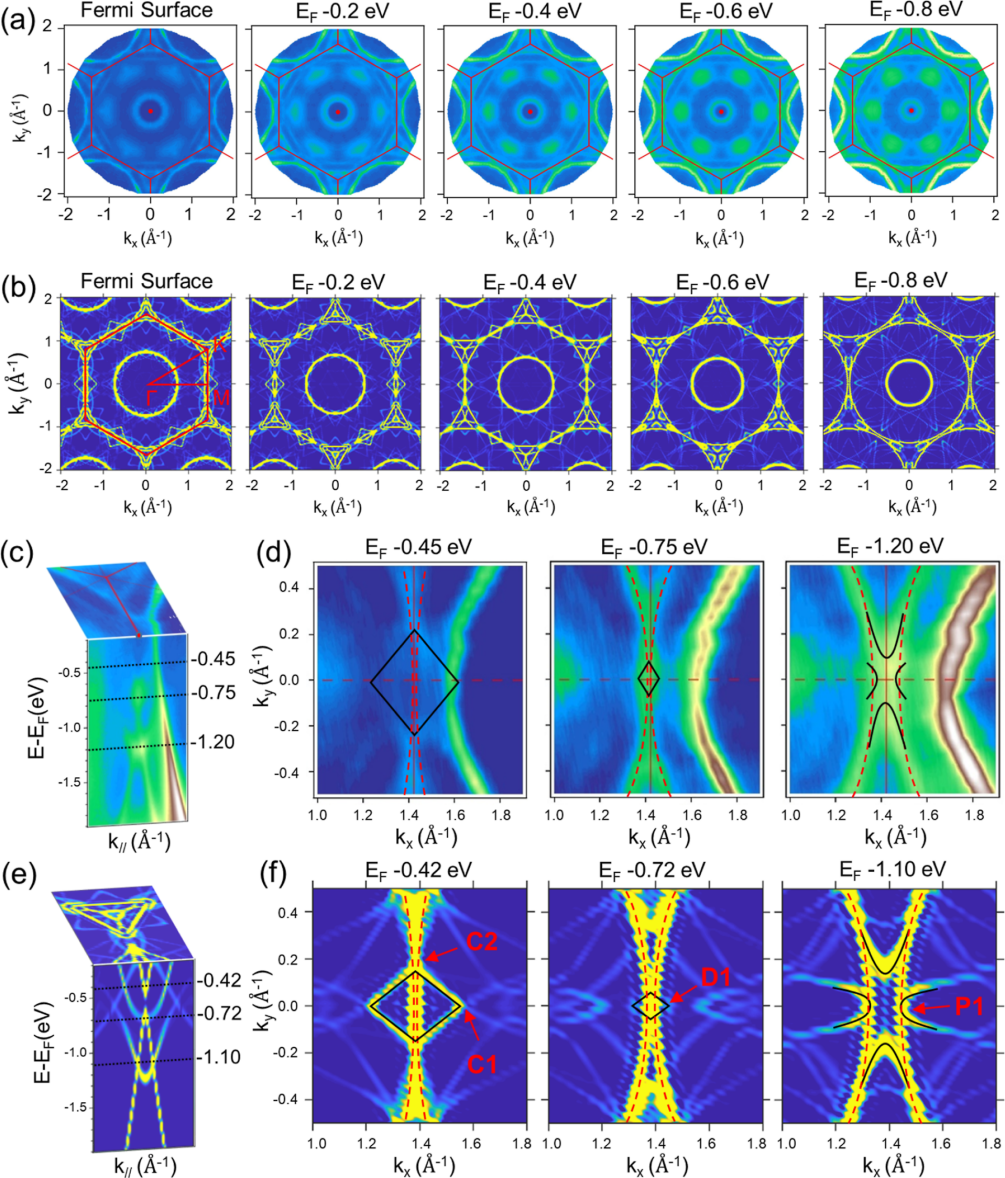
(a) Constant energy full mapping of ARPES spectra ranging from the Fermi level to −0.8 eV binding energy. (b) Isoenergy contour at different binding energies from first-principles calculations for a freestanding β-Sn single layer. Red hexagons represent the BZ of Cu(111) with high-symmetry points marked and labeled. (c) Zoom-in of the energy and momentum scales of the band structure near the M_Cu_ and K_Cu_ point. (d) Zoom in on the map of ARPES spectra at energies −0.45, −0.75, and −1.20 eV around the M_Cu_ point. (e) Constant energy contour connects the electronic band structure near the M_Cu_ and K_Cu_ point. (f) Zoom in on the contours at energies −0.42, −0.72, and −1.10 eV around the M_Cu_ point. The black frame at energies −0.42 and −0.72 eV is the nodal rings. The black frame, black curve and red dashed curve represent the shape of the band structure of the two upper curves and the Dirac cone in Figure 3b on the constant energy contour, respectively.

Another topological transition with the Dirac point labeled with D1 could be also seen in Figure 3a,b. This Dirac-like crossing point is degraded from the type III nodal line,^42^ which is characterized by the crossing line between a cone and a saddle, as plotted schematically in Figure 3d. Both the ARPES (Figure 3a) and DFT (Figure 3b) band structures show that this D1 point consists of a bowl with an upward opening and a saddle with a sharp downward cone-shape dispersion along the M_Cu_–Γ_Cu_ direction and a smooth upward bowl-shape dispersion along the M_Cu_–K_Cu_ direction. Although the intersection of these two energy bands forms the D1 point, they actually intersect with each other over a small ring (Figure 4f) rather than only at a point. However, this ring is indeed small and approximates a quasi-Dirac point as indicated in Figure 3a,b,d. On the other hand, the hyperbolas near P1 point (Figure 3b) intersect into an open curve rather than a closed nodal ring, which can be seen in both the ARPES and DFT contour plots in Figure S8, and is thus not discussed here. It is also worthwhile to note that the gap of ∼0.1 eV at the Dirac-like point around −0.6 eV at M_Cu_ of freestanding β-Sn is induced by strain effect owing to the Cu(111) substrate. This gap is closed with the fully relaxed freestanding β-Sn lattice constants as shown in Figure S9. As for the gap of ∼0.22 eV right below *E*_f_ and ∼0.2 eV at ∼−0.7 eV binding energy around M_Cu_, both originate from SOC and are closed in non-SOC calculations as shown in Figure S10. The lattice distortion-induced gaps are also analyzed in Figure S10.

The decomposed unfolded band structure of the freestanding ML β-Sn (Figure S6) shows that the energy bands near M_Cu_ point are mainly contributed by the p_*xy*_ orbital, while the p_*z*_ orbital only contributes to the upward-opening quadratic energy bands, demonstrating the origin of the gapless nodal line behavior (Figure 3). Similar to reported 2D nodal line materials CuSe,^16^ AgSe,^17^ and Cu_2_Si^18^ that 2D materials naturally have mirror reflection symmetry with respect to the *xy* plane (M_Z_), the mirror reflection symmetry also exists in our single-layer β-Sn. As shown in Figure S6, both the α and β bands of p_*xy*_ character exhibit even parity with positive M_Z_. Whereas the γ band of p_*z*_ character owns odd parity with negative M_Z_. These bands with opposite parities thus lead to gapless crossings at C1 and D1 forming the dual nodal line in β-Sn. Consequently, the two gapless nodal rings observed in this work are protected by mirror reflection symmetry. To further evident that these nodal lines are protected by the mirror reflection symmetry, Figure S7 shows unfolded band structure of β-Sn/Cu. Since the Cu substrate breaks the mirror reflection symmetry, the protected gapless nodal lines at C1 and D1 thus become gaped nodal lines, which provides strong evidence that the dual nodal line observed in this work is protected by the mirror reflection symmetry.

Furthermore, the constant energy full mapping of ARPES spectra ranging from the Fermi level to −0.8 eV binding energy is displayed in Figure 4a, and a zoom-in of the energy and momentum scales of the band structure near the K_Cu_ point probed from ARPES spectra is shown in Figure 4c. According to the Fermi surface in Figure 4a, there are two triangular-shaped states around the K_Cu_ point, and two ellipse-like shape of electron pockets around the M_Cu_ point. These ARPES results show good agreement with the calculated constant energy mapping of the unfolded bands in Figure 4b,e. The DFT/ARPES contour of the Cu(111) slab with and without β-Sn cover layer (Figure S7f/h,g/i, respectively), demonstrate that the outer triangular-shaped state around K_Cu_ is also contributed by Cu(111) substrate, while the inner triangular-shaped state around K_Cu_ is solely β-Sn derived. Moreover, an ellipse-shaped state and a square-shaped state appear around the M_Cu_ point. This ellipse-shaped state and square-shaped state will form a two-electron pocket in the Γ_Cu_–M_Cu_–Γ_Cu_ direction, as shown in Figure 4e. The contour at energies −0.42, −0.72, and −1.1 eV in Figure 4f corresponds to the band intersections C1, C2, D1, and P1 in Figure 3b. The mapping of ARPES for energies −0.45, −0.75, and −1.2 eV in Figure 4d are the results with similar energy band characteristics to those in Figure 4f. The zoom-in contour maps at M_Cu_ from ARPES and DFT (Figure 4d,f, respectively), clearly demonstrate that the C1 and D1 crossings belong to type-I and type-III NLS, respectively, whereas P1 does not. Detailed comparison can be found in Supporting Information Figure S8.

In 3D TNLSM, the projection of node lines onto surfaces forms circles, within which drumhead-like flat surface bands emerge. The drumhead surface states in NLSs are protected by topological invariants defined in the bulk.^43,44^ However, in 2D NLSs, the topological node line does not protect any edge state due to the vanishing codimension of the node line in 2D.^45,46^ Consequently, the edge states are not directly connected to the Dirac points and do not represent a feature of the nodal structure, making their investigation challenging.^47−50^ While ARPES experiments have been employed to study most TNLSM, their limited momentum resolution in the direction perpendicular to the sample surface poses a challenge. Currently, only a few materials have been proposed as 3D TNLSM,^14,51−55^ while the realization of topological nodal-line semimetals in 2D materials is limited due to the requirement of high symmetry and lower SOC.^56,57^ There have been some theoretical predictions suggesting that certain 2D materials can exhibit a TNLSM phase under applied strains or electric fields.^12,13^ So far the only experimental realizations can be found in the aforementioned CuSe,^16^ AgSe,^17^ and Cu_2_Si^18^ with similar structural character and type-I nodal line behavior. Furthermore, all three of these gapless nodal rings become gapped as SOC is included. In this work, the measured band structure and performed DFT calculations on a single layer β-Sn/Cu(111) sample reveal the coexistence of type I and type III gapless nodal line behaviors. Our STM–ARPES–DFT combined work of ML β-Sn hence serves as a new-type 2D dual-NLS. This discovery provides a valuable platform for exploring intriguing phenomena and potential applications related to TNLSM and topological superconductors in the 2D limit.

## Conclusions

In this work, we successfully prepared single layers of α-Sn(111) and β-Sn(001) on a Cu(111) substrate. By combining STM, ARPES, and DFT calculations, we elucidate the structural and electronic phase transitions in ML Sn from honeycomb α-Sn to cubic β-Sn as the Sn coverage increases. The good agreement between DFT calculations and STM as well as ARPES results provides an overall consistent structural and electronic picture. The electronic structure exhibits a topological transition from 2D topological insulator α-Sn to 2D TNLSM β-Sn with type-I and type-III nodal lines coexisting in ML β-Sn. Interestingly, we also observe extremely free-standing-like electronic structures of β-Sn/Cu(111), which is a rare and unexpected phenomenon, especially in a metal layer/metal substrate system. Our STM–ARPES–DFT combined discovery of a new-type 2D dual-nodal line material demonstrates the high potential of ultrathin β-Sn films as a platform for exploring the electronic properties of 2D TNLSM and 2D topological superconductors, such as few layer superconducting Sn in lateral contact with topological nodal line single layer β-Sn.

## Methods

### Scanning Tunneling Microscopy

The Sn/Cu(111) sample was prepared in the ultrahigh vacuum (UHV) chamber with a base pressure of 1 × 10^–10^ mbar. To get a clean substrate surface, Cu(111) single crystal substrate was sputtered with a low Ar^+^ ion energy of 0.5 keV for several cycles. Subsequently, the substrate was annealed up to 700 K and cooled down to 80 K on the cooling manipulator. Next, the Sn was evaporated onto the cold Cu(111) substrate to form single-atomic-layer α-Sn and β-Sn thin film. Finally, the sample was immediately transferred to low-temperature STM from Unisoku Co. Ltd. (operation temperature *T* ≈ 4.2 K). The topographic images were acquired in the constant-current mode.

### Angle Resolved Photoemission Spectroscopy

The ARPES experiment was conducted at beamline BL21B1 in the Taiwan Light Source (TLS), in the National Synchrotron Radiation Research Center (NSRRC). To prepare the Cu(111) single crystal surface, it was cleaned repeatedly in a UHV environment using sputtering and annealing cycles until a sharp LEED pattern and the sharp surface state of Cu(111) were observed. Sn atoms were deposited onto the surface using a Knudsen cell with a calibrated deposit rate, measured by a quartz thickness monitor. The deposition took place at 80 K in the upper preparation chamber, resulting in the formation of single-layer α-Sn and β-Sn phases on the Cu(111) surface, which were confirmed by LEED patterns as the amount of Sn deposition increased. The ARPES data were collected in an UHV chamber equipped with a hemispherical analyzer (Scienta R4000) with an ±15° collecting angle and 0.5° angular resolution. All spectra were recorded at 80 K and base pressure of 6.1 × 10^–11^ Torr, with incident photon energies ranging from 64 to 74 eV. The overall energy resolution was better than 24 meV.

### Density Functional Theory

First-principles calculations were performed using the Vienna Ab Initio Simulation Package (VASP)^58−60^ based on density functional theory (DFT). The projector augmented wave^61^ pseudopotential with the generalized gradient approximation in the Perdew–Burke–Ernzerhof^62^ form for exchange–correlation potential was used. The vacuum layer thickness of 25 Å was adopted in the slab model calculations with the plane wave cutoff energy of 400 eV. The slab model of the  β-Sn supercell (red rectangular) in Figure S5a used in the calculations is constructed from the common lattice of the ML  β-Sn supercell (black rhombus) containing 3 Sn atoms and the 6-layers 1 × 1 Cu(111) (blue rhombus) substrate. We then performed geometrical optimization using 6 × 18 × 1 Monkhorst–Pack *k*-mesh over the 2D BZ until the total energy and residual atomic force are less than 10^–6^ and 0.005 eV/Å, respectively. The electronic structure calculations of the relaxed lattice structure using 6 × 18 × 1 *k*-mesh were carried out with SOC included self-consistently. Based on this relaxed slab model, we also performed self-consistent SOC calculations of freestanding ML  β-Sn(001) for comparison. To be compared with our ARPES results, the electronic band structure of the ML β-Sn(001) with and without Cu(111) substrate were unfolded onto the BZ of Cu(111) 1 × 1 unit cell via the BandUp code.^63,64^

## Supporting Information Available

STM and LEED measurements of β-Sn(001) ML; ARPES measurements of β-Sn(001) ML; and DFT 2D nodal line electronic structure calculations of β-Sn(001) ML (PDF)
