# Supplementary material for: Dual Dirac Nodal Line in Nearly Freestanding Electronic Structure of β-Sn Monolayer
Source: ACS Nano. 2024 Aug 1;18(32):20990–8. doi: 10.1021/acsnano.4c01322 (PMC11328162; doi:10.1021/acsnano.4c01322)
Supplement: Supplementary file 1 — nn4c01322_si_001.pdf [file nn4c01322_si_001.pdf]

Supporting Information for

Dual Dirac Nodal Line in Nearly Freestanding Electronic Structure of β-Sn Monolayer

Ye-Shun Lan[1,*], Chia-Ju Chen[1,*], Shu-Hua Kuo[2,*], Yen-Hui Lin[1], Angus Huang[1,3,4], Jing-Yue Huang[2], Pin-Jui Hsu[1,5,†], Cheng-Maw Cheng[2,6,7,‡], and Horng-Tay Jeng[1,4,8,‡†]

[1]Department of Physics, National Tsing Hua University, Hsinchu 30013, Taiwan

[2]National Synchrotron Radiation Research Center, Hsinchu 30076, Taiwan

[3]Center for Theory and Computation, National Tsing Hua University, Hsinchu 30013, Taiwan

[4]Physics Division, National Center for Theoretical Sciences, Taipei 10617, Taiwan

[5]Center for Quantum Technology, National Tsing Hua University, Hsinchu 30013, Taiwan

[6]Department of Electrophysics, National Yang Ming Chiao Tung University, Hsinchu 30010, Taiwan.

[7]Department of Physics, National Sun Yat-sen University, Kaohsiung 80424, Taiwan

[8]Institute of Physics, Academia Sinica, Taipei 11529, Taiwan

[*] These authors contributed equally to this work.

Corresponding authors. E-mail:[†]pinjuihsu@phys.nthu.edu.tw;[‡]makalu@nsrrc.org.tw;[‡†]jeng@phys.nthu.edu.tw

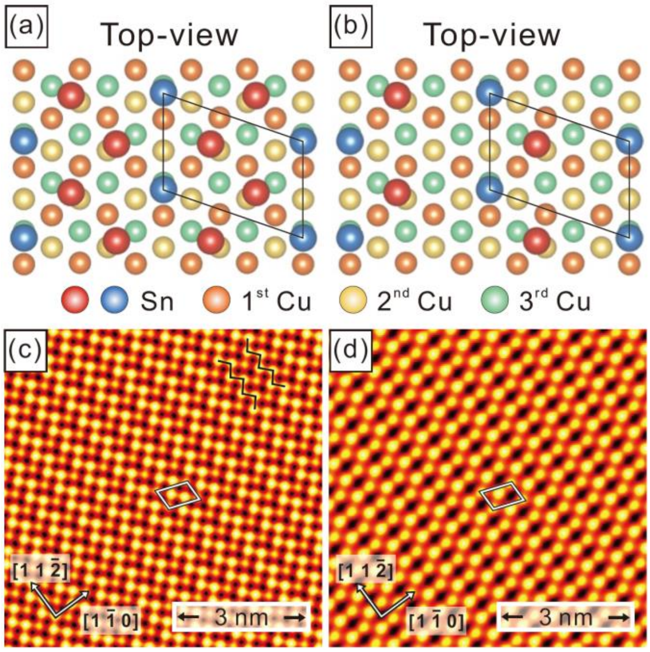

Figure S1. Comparison of different structure models and the corresponding simulated STM images. Fig. S1(a) is the top view of the (2113) HC-Sn structure model with three Sn atoms per unit cell, and Fig. S1(b) is the top view of the (2113) Sn structure model with two Sn atoms per unit cell as proposed in previous LEED study[1]. The corresponding STM simulated image of Fig. S1(a) has been shown in Fig. S1(c), where the white rhombus frame marks the unit cell consisting of three Sn and the characteristic periodic zigzag patterns (black stripes) are in good agreement with the experimental results from Fig. 2(a). In order to compare the structure model in the Fig. S1(b) from the LEED measurements reported before[1], we have also carried out the STM simulations accordingly and the results are shown in the Fig. S1(d). The unit cell with two Sn atoms has been identified by the white rhombus frame, and one can clearly see the absence of periodic zigzag pattern in stark contrast to the results in the Fig. S1(c) and Fig. 2(a). Therefore, the (2113) HC-Sn structure model with three Sn atoms per unit cell agrees better with our experimental results from Fig. 2(a).

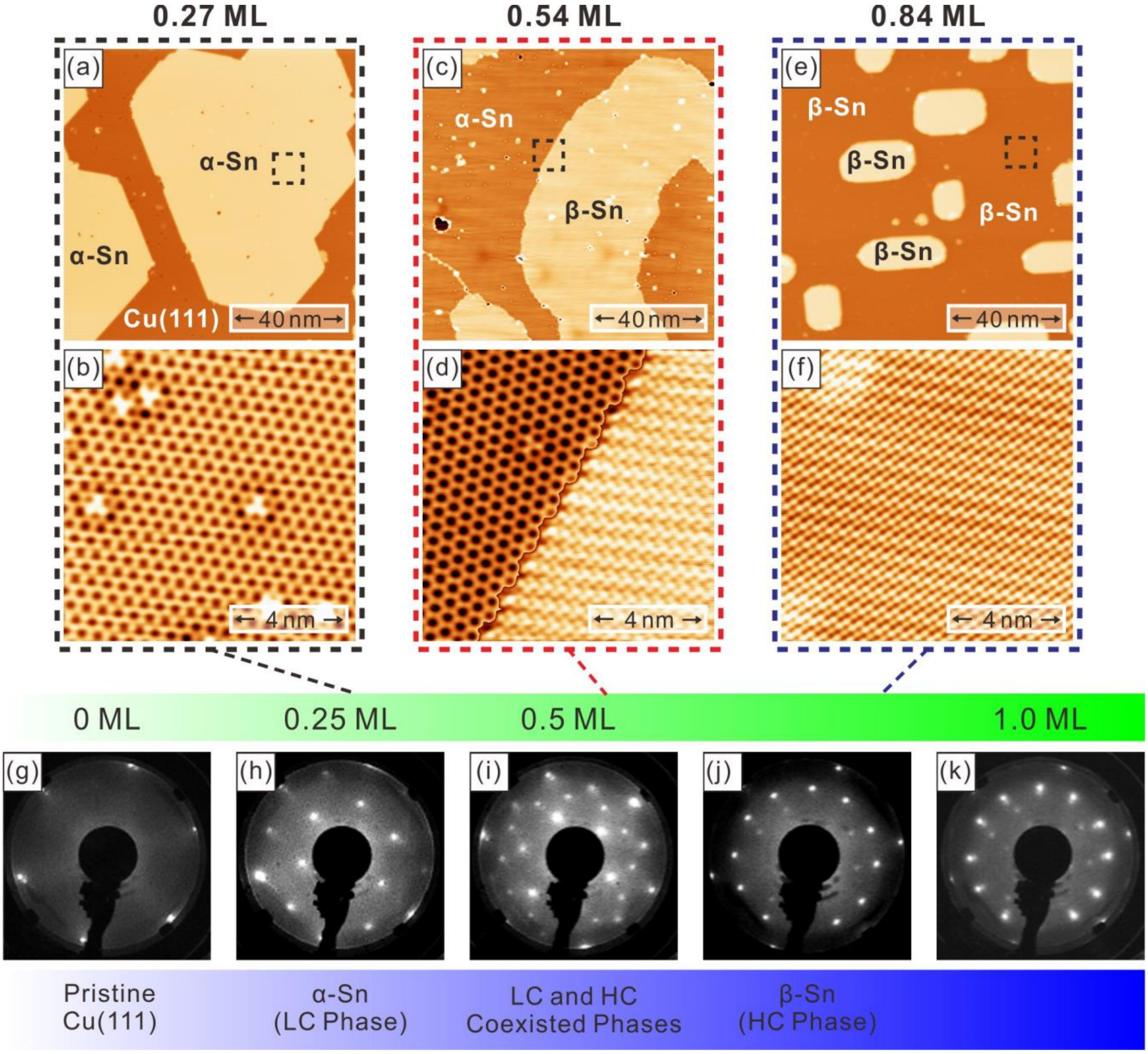

Figure S2. To identify α- and β-Sn structures on Cu(111), three distinct experimental measurements have been carried out. First of all, we have applied STM to obtain not only topographic overview, but also atomic resolution image of Sn thin layer/Cu(111) at different Sn surface coverages. As shown in Fig. S2(a), STM image represents topographic overview of submonolayer Sn deposited onto Cu(111). In Fig. S2(b), corresponding atomically-resolved image, i.e., acquired from the black dashed square in Fig. S2(a), reveals the α-Sn with honeycomb lattice. Since honeycomb-structured α-Sn has a p(2x2) unit cell on Cu(111), the Sn surface coverage of 0.27 ML can be deduced. By increasing the amount of Sn coverage, the high-coverage (HC) Sn phase, i.e., β-Sn, appears and coexists with honeycomb-structured α-Sn in topographic overview of Fig. S2(c), where atomic resolution image supports the coexistence shown in Fig. S2(d). While the β-Sn exhibits a $\sqrt{3}\times\sqrt{7}$ supercell containing 3 Sn atoms, one can derive the Sn coverage thickness of 0.54 ML. When the amount of Sn coverage has been further increased, in addition to the β-Sn monolayer, the β-Sn islands with an apparent height of 3 atomic layers have also emerged from the topographic overview in Fig. S2(e). The atomic lattice of the β-Sn monolayer has been displayed in Fig. S2(f) and the Sn surface coverage of 0.84 ML can be obtained.

Secondly, the evolution of LEED patterns from Fig. S2(g) to (k) with varied Sn surface coverage has been observed. A 2x2 LEED pattern relative to Cu (111) substrate with 0.25 ML surface coverage indicates the α-Sn phase on Cu(111). With increasing Sn surface coverage, a coexistence of dual phases of α-Sn and β-Sn appears on LEED pattern, and then further changes to β-Sn phase only when the Sn coverage continues increasing.

Lastly, we have also performed the ARPES measurements on α- and β-Sn, respectively. From the results shown in following Fig. S3, one can clearly see the large difference is the band structures of α- and β-Sn, offering another important perspective to distinguish the α-Sn from the β-Sn on Cu(111) in the submonolayer range, i.e., below one full atomic layer surface coverage.

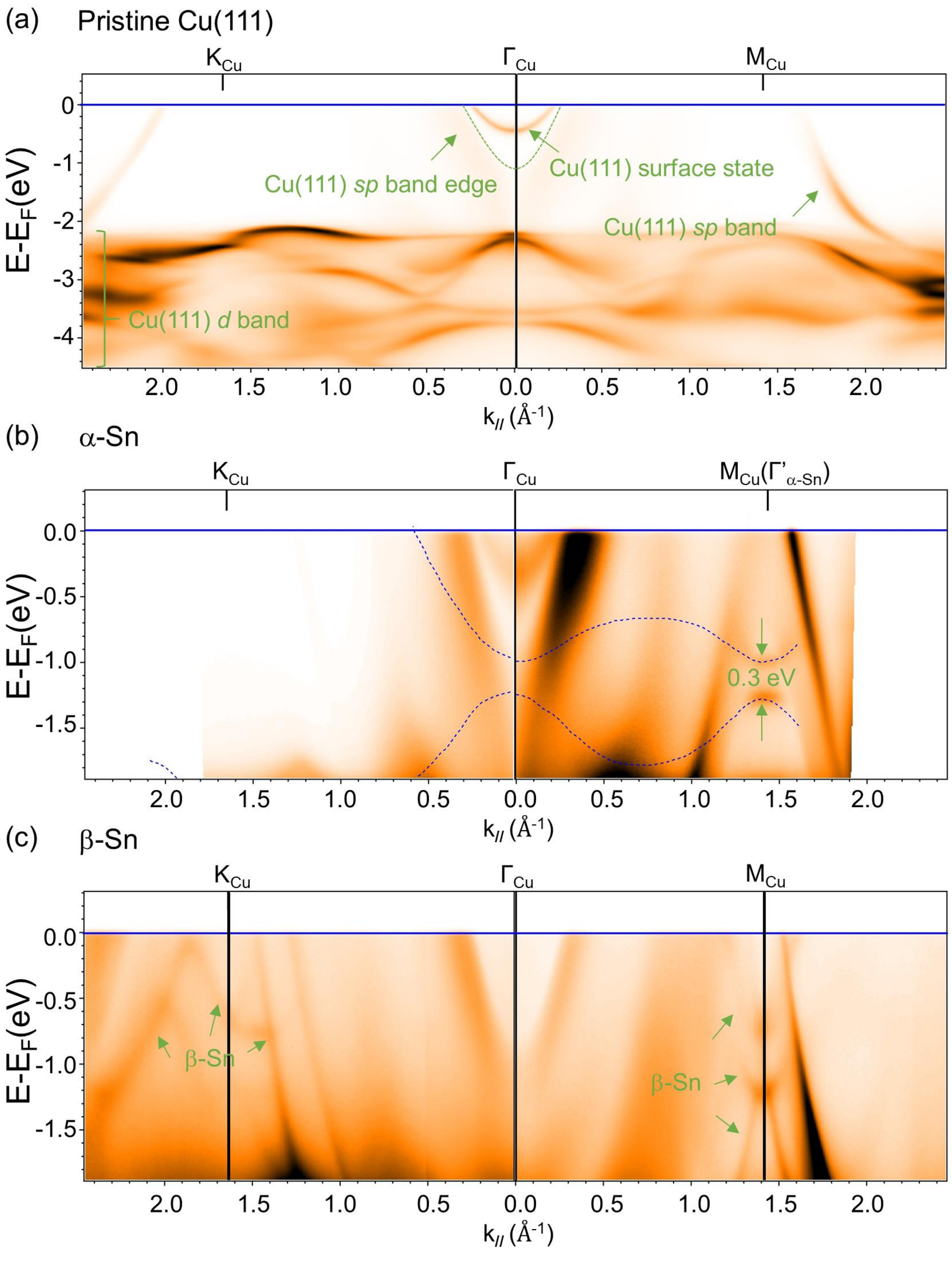

Figure S3. The comparison of the band structures of pristine Cu(111), α-Sn and β-Sn, correspond to figure S3 (a),(b) and (c). The overlapped band dispersion in (b) is extracted from the reported work[2], show a good consistency of presented work. In figure S3 (c), the Sn-derived bands on β-Sn can be significantly distinct from the measured band structures around the K_{Cu} and M_{Cu} points.

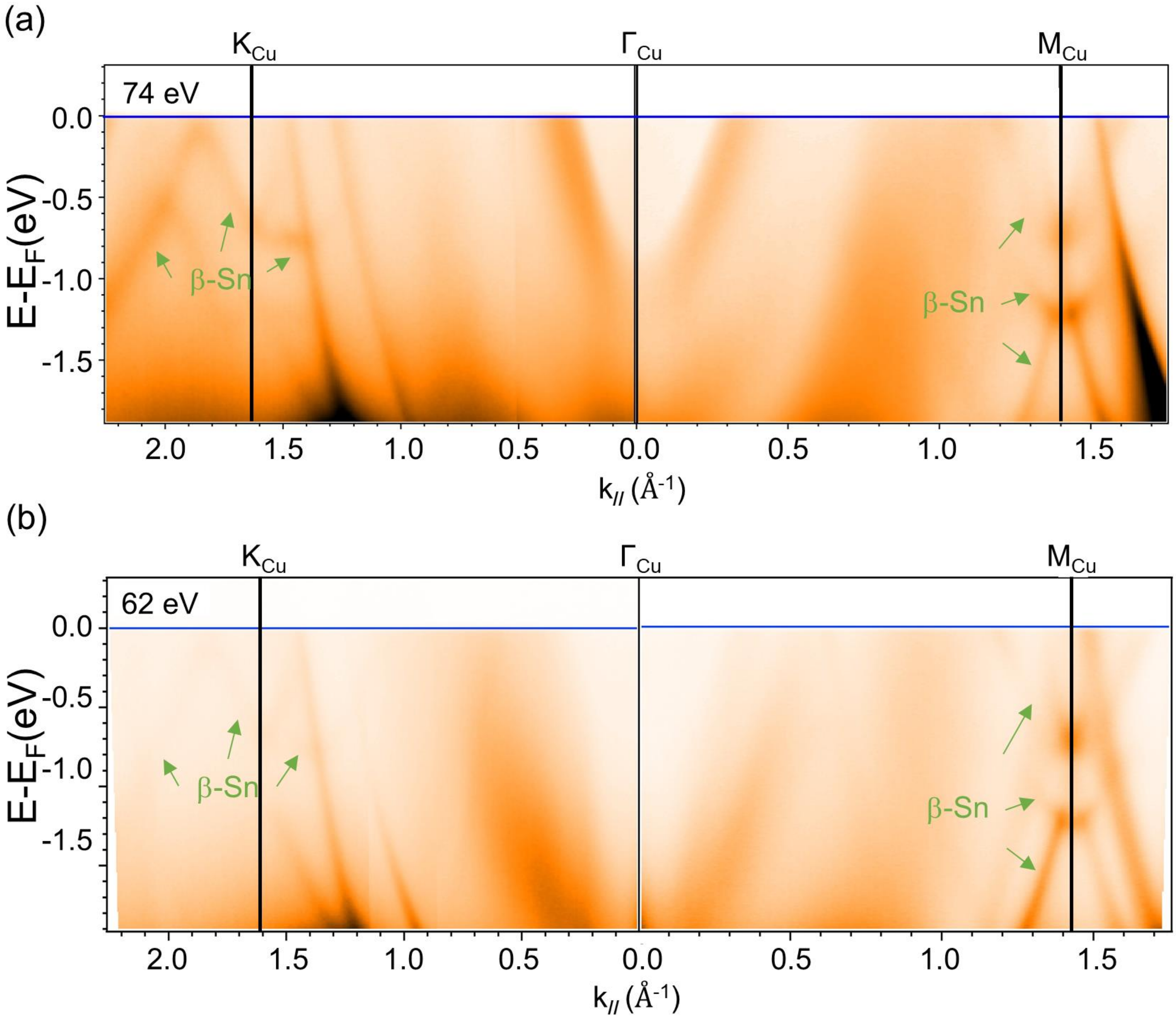

Figure S4. The photon energy dependent of ARPES spectra recorded at 74 eV(a) and 62 eV(b). The photon energy *hv* in the ARPES experiment is correlated to the k_z of *k*-space. The contribution of β-Sn near the high symmetry point K_{Cu} and M_{Cu} is similar between Fig.S4(a) and (b) except the photoemission intensity due to the matrix element effect. The fact that the β-Sn moiety does not change when k_z is different indicates that the measured band structure is a two-dimensional behavior and derived from β-Sn.

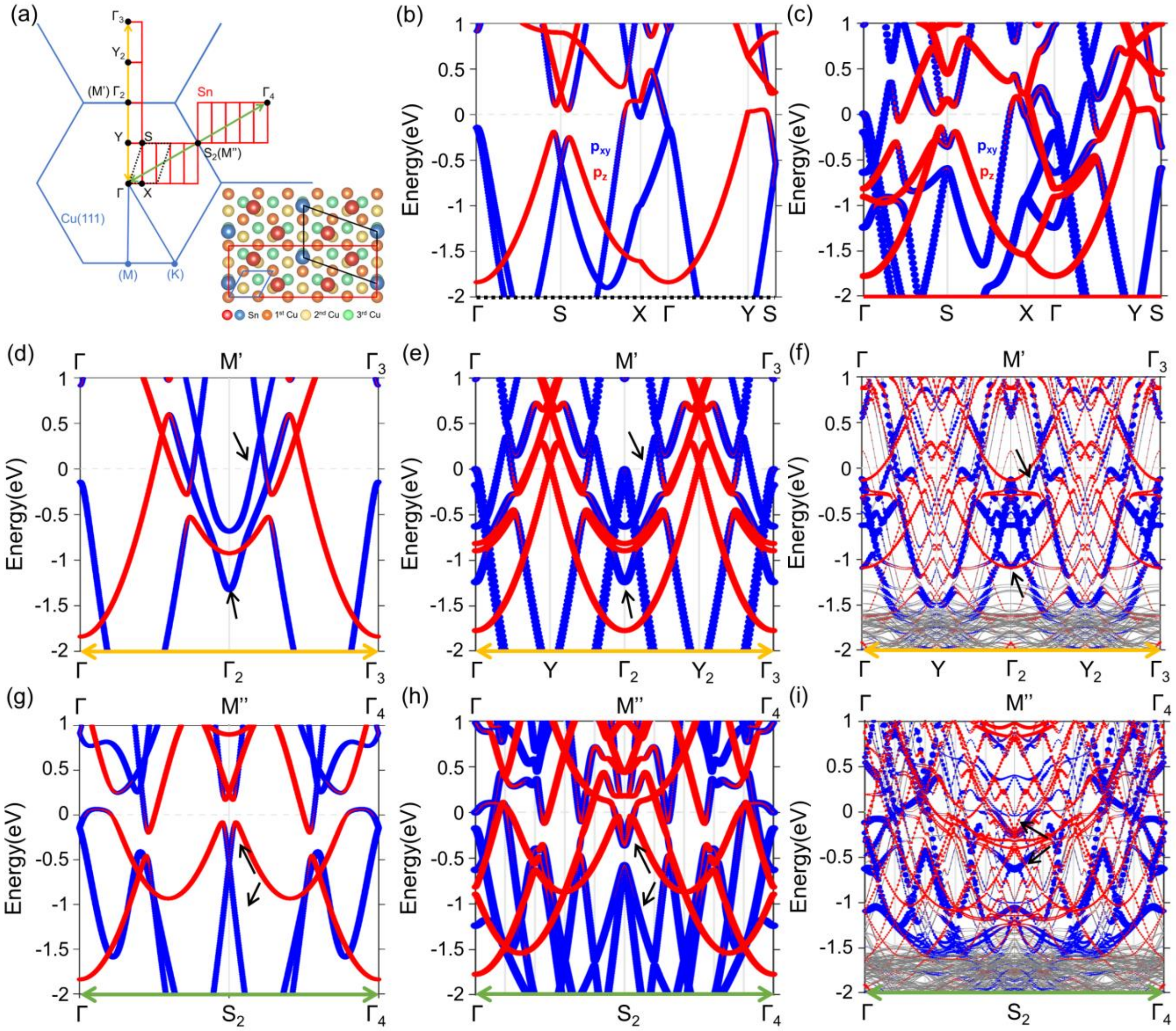

Figure S5. Electronic band structures of β-Sn and β-Sn/Cu(111) structure with SOC. (a) 2D BZ and lattice structure of monolayer β-Sn on 6-layer Cu(111) substrate. Blue rhombus, black parallelogram, and red rectangle indicate the 1x1 Cu(111) substrate unit cell, the monolayer $\sqrt{3}\times\sqrt{7}$ β-Sn supercell, and their common lattice of $\sqrt{3}\times 5$ β-Sn supercell, respectively. Blue hexagon, black parallelogram, and red rectangle BZ correspond to the Cu 1x1 unit cell, the monolayer $\sqrt{3}\times\sqrt{7}$ β-Sn supercell, and the common $\sqrt{3}\times 5$ β-Sn supercell, respectively. (b)(d)(g) DFT band structures using the surface unit cell of β-Sn containing 3-Sn atoms (black parallelogram) along the high symmetry lines in BZ following the red rectangle, orange and green lines, respectively, as indicated in (a). (c)(e)(h) DFT band structures of freestanding β-Sn in the 6-Sn red rectangle supercell along the high symmetry lines in BZ through the red rectangular, orange and green lines, respectively, as indicated in (a). The size of the blue and red spheres denote the Sn $p_{x,y}$ and p_z components, respectively. Black arrows in (c) and (d) indicate bands relevant to the unfolded band structure around the M_{Cu}-point. (f)-(i) DFT band structures of the β-Sn/Cu(111) supercell (red rectangle). For comparison with ARPES results, the unfolded band structures in Figs. S6,7,9,10 go through the path M_{Cu}-K_{Cu}-Γ_{Cu}-M_{Cu} as indicated by the blue triangle in (a).

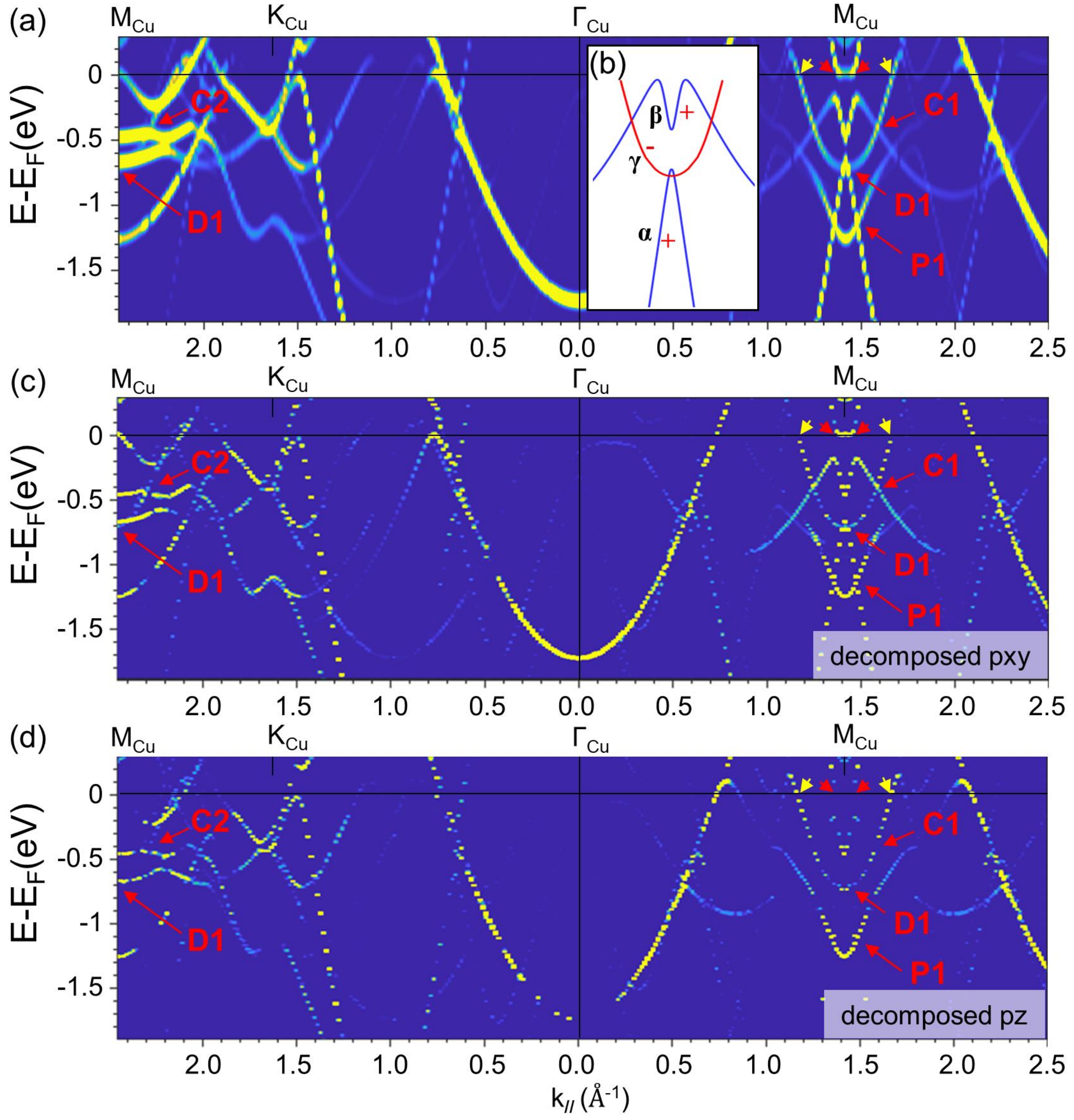

Figure S6. (a) Unfolded band structure of freestanding β-Sn monolayer with SOC included. (b) Schematic plot of the three bands α, β , γ related to the two nodal lines near the M_{Cu} point. Both the α and β bands of p_{xy} character (c) exhibit even mirror parity with M_Z eigenvalue +1 as indicated by the plus sign. The γ band of p_z character (d) owns odd mirror parity with M_Z eigenvalue -1 (minus sign). (c) p_{xy}-decomposed unfolded band structure of (a). (d) p_z-decomposed unfolded band structure of (a). Around the high symmetry point M_{Cu}, p_z orbital contributes mostly in the parabolic bands, while p_{xy} components can be found in all bands. Similar to the reported 2D nodal line materials CuSe[3], AgSe[4] and Cu2Si[5], the dual Dirac nodal line observed in this work is protected by the mirror reflection symmetry. This is further confirmed in Fig. S7 by analyzing the Cu-substrate effect on the unfolded β-Sn bands.

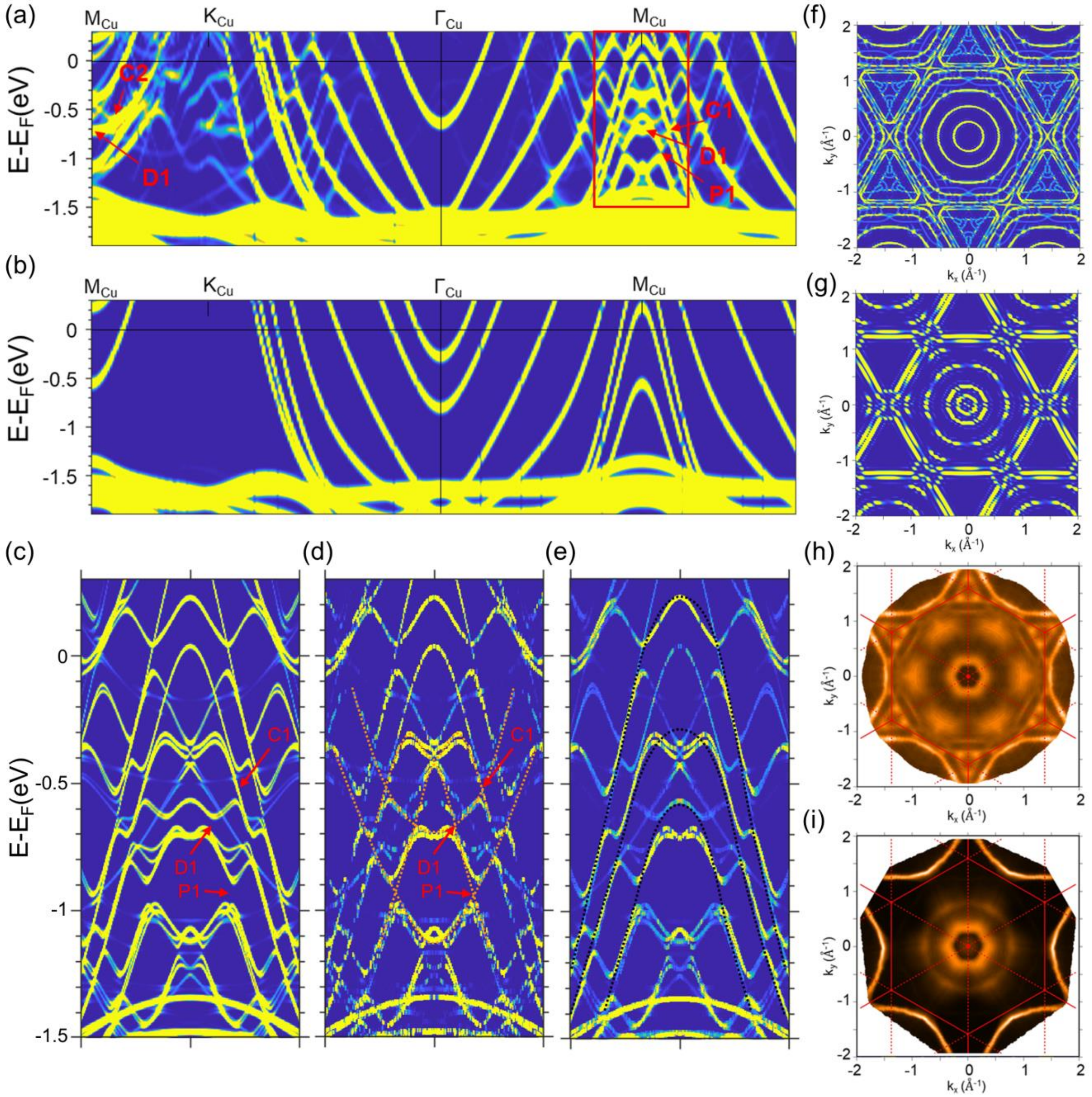

Figure S7. (a) The calculated unfolding band structure of β-Sn(001)/6-layer Cu(111) over the surface BZ of Cu(111). Red arrows indicate band crossings D1, C1, and C2. E_f is at the zero energy. (b) The calculated unfolding band structure of 6-layers of Cu(111). E_f is at the zero energy. In comparison with bands in (a), the β-Sn-derived bands can be identified. (c) High-resolution unfolded bands in the red rectangle in (a). (d) Sn contributions of (c). The orange dotted lines indicate the Sn-derived bands related to nodal lines corresponding to the α, β , γ bands in Fig. S6. (e) Cu contributions of (c). The black dotted lines indicate the Cu-derived bands. The Cu substrate breaks the mirror reflection symmetry, the protected gapless nodal lines at C1 and D1 become gaped nodal lines as indicated by the red arrows in (c). An extra band accidentally passing through the nodal gap at C1 in (c), it is a Cu-substrate-derived band as shown in (e). In comparison with Fig. S6, the dual nodal line observed in this work is protected by the mirror reflection symmetry. (f) The DFT unfolded contour of β-Sn(001)/Cu(111) at E_f. (g) The DFT unfolded contour of 6-layers of Cu(111) at Fermi level. (h) The constant energy mapping of β-Sn(001)/Cu(111) at E_f, the picture is plotted with a three-fold symmetry

from the ARPES raw data. (i) The constant energy mapping of Cu(111) at E_f, the picture is plotted with a three-fold symmetry from the ARPES raw data. It can be seen that the outer triangular-shaped state around K point is also contributed by the Cu substrate, while the inner triangular-shaped state is derived solely from β-Sn.

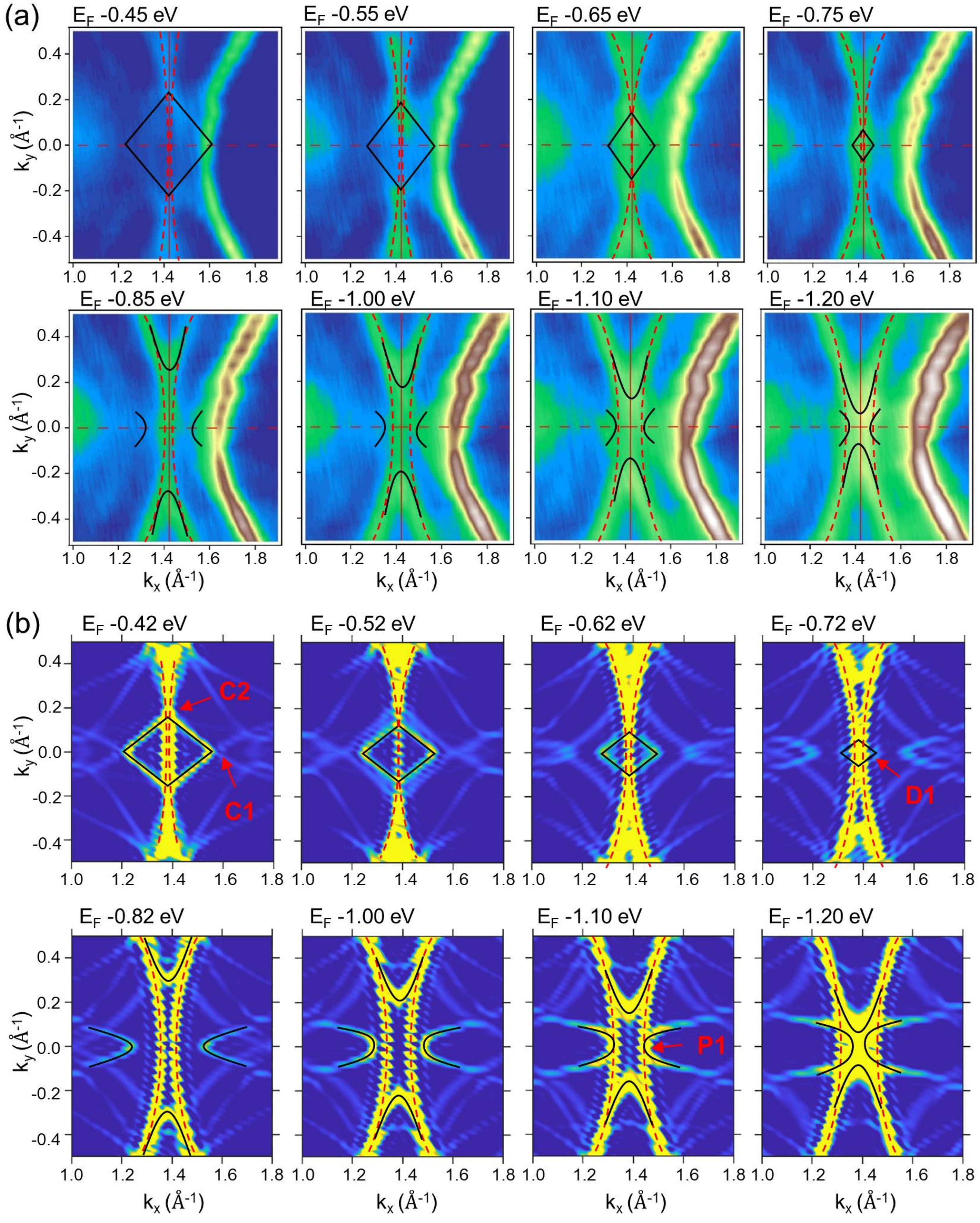

Figure S8. (a) Zoom in on the map of the ARPES spectrum around the M_{Cu} point at energies from -0.45 eV to -1.20 eV. (b) Zoom in the contour based on theoretical calculation around the M_{Cu} point at energy from -0.42 eV to -1.20 eV. The black frame, black curve and red dashed curve represent the shape of the band structure of the two upper curves and the Dirac cone in Fig.3(b) on the constant energy contour, respectively. From energy -0.42 eV to -0.62 eV, the red dashed curve (upper Dirac cone) will slowly approach M_{Cu} point, and from energy -0.62 eV to -1.20 eV, the red dashed curve (lower Dirac cone) will gradually move away from M_{Cu} point. In (b), from energy -0.42 eV to -0.72

eV, the black frame begins to shrink, and forms a small nodal ring (D1) with the red dashed curve (lower Dirac cone) at -0.72 eV. In (a), from energies -0.45 eV to -0.65 eV, although not as clear as in theory, there is a cluster of similar regions that gradually shrink in the black frame. This means that the nodal ring energy of C1-C2-C1-C2-C1 is close to −0.45 eV, and C1 is covered by the edge band of bulk Cu. In (b), from energy -0.82 eV to -1.20 eV, the black curves begin to close M_{Cu} point, and cross P1 with the red dashed curve (lower Dirac cone) at -1.10 eV. In (a), the black curve at energy -0.85 eV is not clear. When the energy goes from -0.85 eV to -1.20 eV, the color under the black curve becomes brighter.

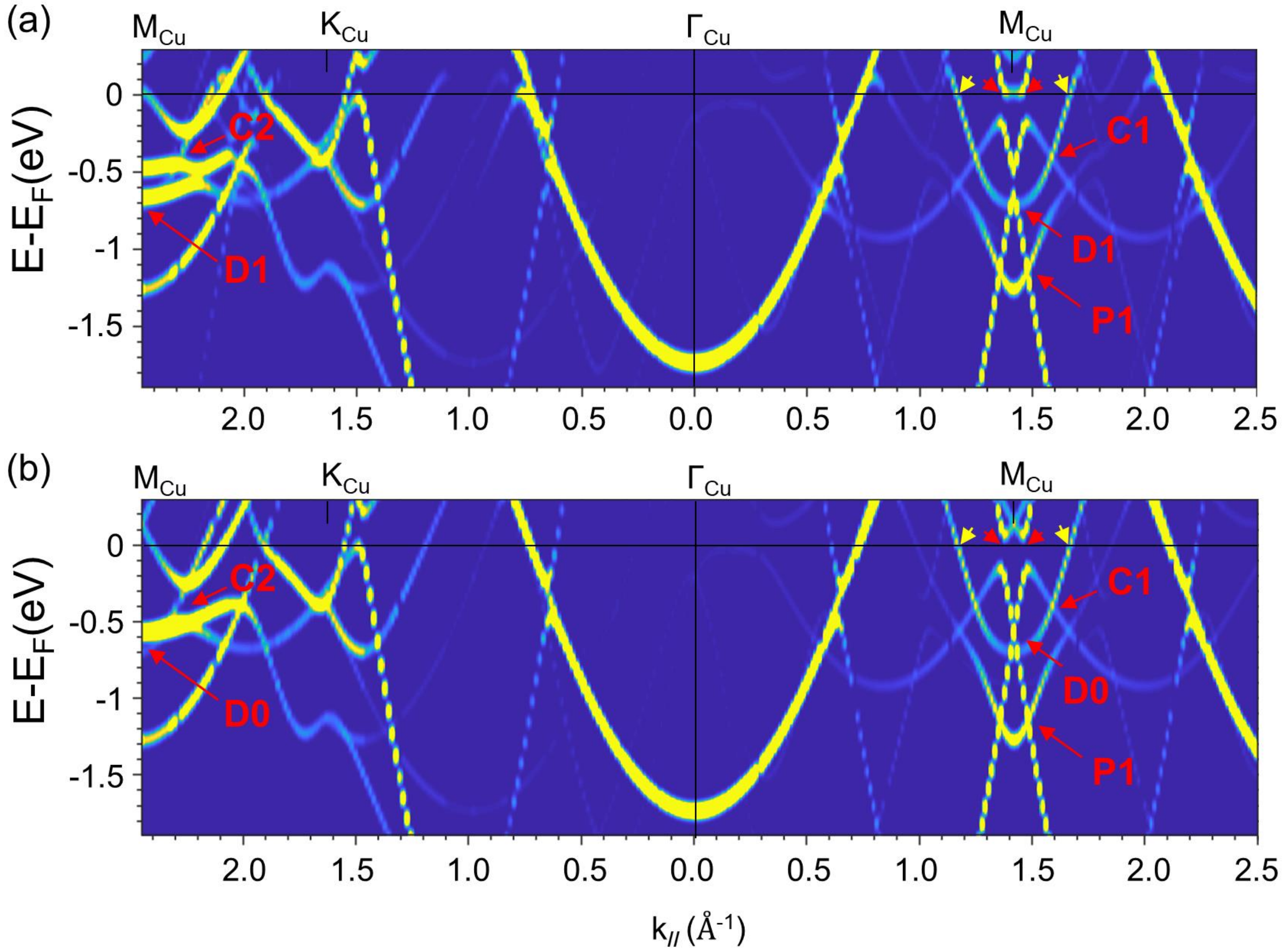

Figure S9. (a) Unfolded band structure of freestanding β-Sn(001) monolayer with SOC using the lattice constant a, b matching the Cu(111) substrate. (b) Unfolded band structure of freestanding β-Sn(001) monolayer with SOC using the fully relaxed lattice constants a, b. The D0 red arrow in (b) indicates the Dirac point, which turns into a strain-induced gap in (a).

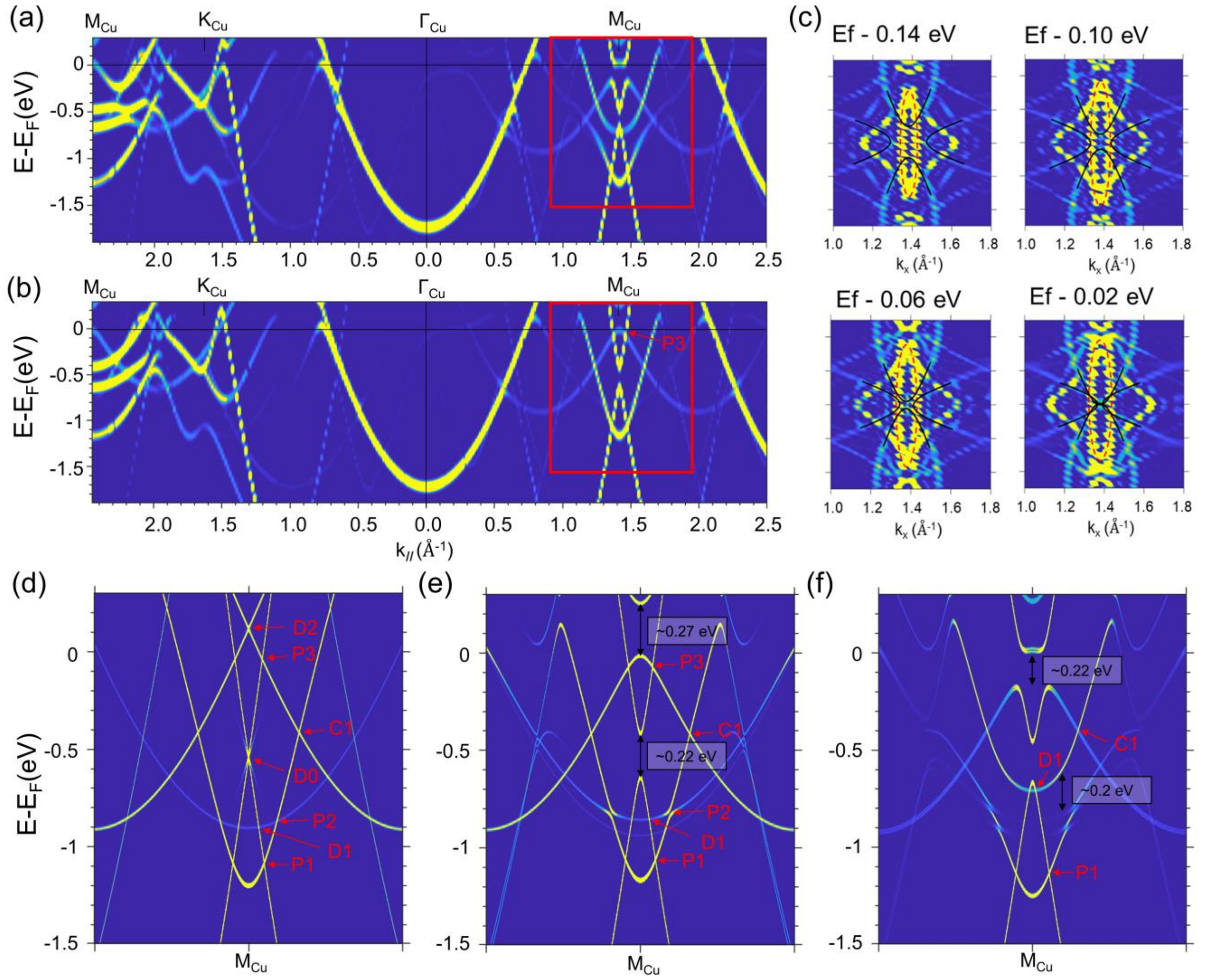

Figure S10. (a) Unfolded band structure of freestanding β-Sn(001) monolayer with SOC included. (b) Unfolded band structure of freestanding β-Sn(001) monolayer with SOC excluded. Red arrows indicate the SOC-induced gap. (c) The contour around the M_{Cu} point in energy from -0.02 eV to -0.14 eV. The red dashed curve and black curve represent the band structure of the upper Dirac cone and the crossing band, respectively, as indicated by the red arrow in (b). These energy bands cross at -0.1 eV, but this cross is not a nodal line as can be seen from the energy-dependent contours. Unfolded band structure around the M_{Cu} point for undistorted freestanding β-Sn without SOC (d), distorted freestanding β-Sn with the lattice structure given from the geometrically optimized β-Sn/Cu substrate without SOC (e), and distorted freestanding β-Sn with SOC (f). (d) shows gapless Dirac-like point D0, nodal line C1, and nodal line D1 without distortion and SOC. (e) shows distortion-induced gap of ~0.22 eV and ~0.27 eV emerging at D0 and D2, respectively. A small gap in the meV order is also opened at P2 due to the lattice distortion. (f) shows the SOC-induced band gap of ~0.2 eV and ~0.22 eV at P2 and P3, respectively, demonstrating the degeneracy breaking around the M_{Cu} point owing to SOC.

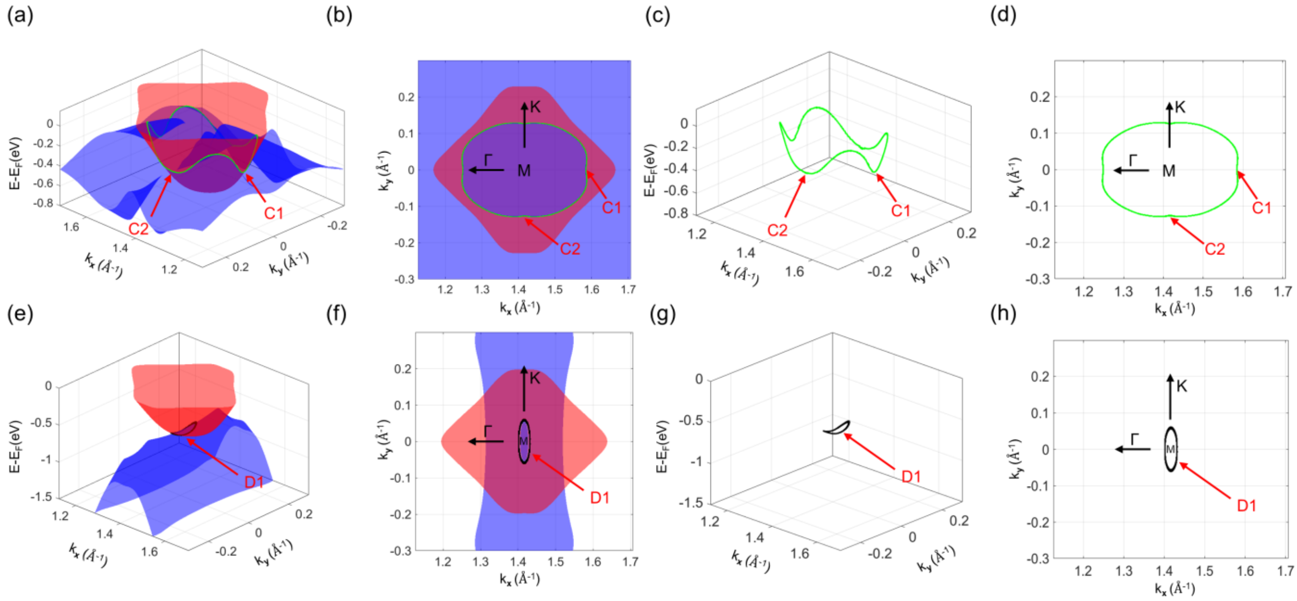

Figure S11. (a)(e) Energy dispersion of the dual nodal line over the 2D BZ. The nodal lines (green in (a) and black in (e)) are given from the crossings of the blue and red bands. (c)(g) show only the nodal lines in (a)(e), respectively, for a clearer view. (b)(f) Precise locations of the nodal lines in the 2D BZ. For clarity, (d)(h) show the nodal lines in the 2D BZ only.

REFERENCES:

(1) Liang, X.; Deng, J.-H.; Fan, L.-J.; Yang, Y.-W.; Luh, D.-A. Nonalloying Surface Reconstructions of Ultrathin Sn Films on Cu(111) Investigated with LEED, XPS, and Photoelectron Extended Fine Structure analysis. *Phys. Rev. B* **2011**, *84* (7), 075406.

(2) Deng, J.; Xia, B.; Ma, X.; Chen, H.; Shan, H.; Zhai, X.; Li, B.; Zhao, A.; Xu, Y.; Duan, W.; et al. Epitaxial Growth of Ultraflat Stanene with Topological Band Inversion. *Nat. Mater.* **2018**, *17* (12), 1081-1086.

(3) Gao, L.; Sun, J.-T.; Lu, J.-C.; Li, H.; Qian, K.; Zhang, S.; Zhang, Y.-Y.; Qian, T.; Ding, H.; Lin, X.; et al. Epitaxial Growth of Honeycomb Monolayer CuSe with Dirac Nodal Line Fermions. *Adv. Mater.* **2018**, *30* (16), 1707055.

(4) Lu, J.; Gao, L.; Song, S.; Li, H.; Niu, G.; Chen, H.; Qian, T.; Ding, H.; Lin, X.; Du, S.; et al. Honeycomb AgSe Monolayer Nanosheets for Studying Two-dimensional Dirac Nodal Line Fermions. *ACS Appl. Nano Mater.* **2021**, *4* (9), 8845-8850.

(5) Feng, B.; Fu, B.; Kasamatsu, S.; Ito, S.; Cheng, P.; Liu, C.-C.; Feng, Y.; Wu, S.; Mahatha, S. K.; Sheverdyaeva, P.; et al. Experimental Realization of Two-Dimensional Dirac Nodal Line Fermions in Monolayer Cu2Si. *Nat. Commun.* **2017**, *8* (1), 1007.
